# Impact of peer interaction on maternal health service utilization in rural northwest China

**DOI:** 10.3389/fpubh.2024.1495667

**Published:** 2025-01-08

**Authors:** Liuchun Xiang, Dan Li, Junhao Wu, Jun Chen, Jie Yang, Haisong Nie

**Affiliations:** ^1^Center for Experimental Economics in Education, Faculty of Education, Shaanxi Normal University, Xi’an, China; ^2^United Graduate School of Agricultural Science, Tokyo University of Agriculture and Technology, Tokyo, Japan; ^3^Institute of Agriculture, Tokyo University of Agriculture and Technology, Tokyo, Japan

**Keywords:** peer interaction, pregnant and postpartum women, maternal health service, propensity score matching, rural area

## Abstract

**Background:**

Effective use of health services by pregnant and postpartum woman (PPWs) is crucial to maternal and child health. Most maternal deaths are attributed to inadequate maternal health services, especially in rural areas. As a vulnerable group, rural PPWs can effectively prevent and reduce maternal and infant health risk factors through whole-process health management and ensure the health and safety of mothers and infants. Therefore, improving the utilization rate of rural maternal health services is a key issue that needs to be addressed urgently. This study aimed to explore the influence of peer interaction on the utilization of maternal health services in rural areas and the mediating effect of maternal health service knowledge.

**Methods:**

Based on cross-sectional data of 821 PPWs in rural northwest China. This study used propensity score matching (PSM) to analyze the effects of peer interaction (PI) on maternal health service utilization, including maternal system management rate (Y1), prenatal screening rate (Y2), and postpartum visit rate (Y3). In addition, the mediating role of maternal health service knowledge between peer interaction and health service utilization was empirically tested.

**Results:**

The findings highlight the important role of peer interaction in improving the utilization rate of maternal health services in rural northwest China. The study found that peer interaction significantly improved the maternal system management rate, prenatal screening rate, and postpartum visit rate. At the same time, peer interaction enhances knowledge of maternal health services, which plays a key role in improving maternal health behaviors.

**Conclusion:**

Through experience sharing and knowledge exchange among peers, understanding of health services can be enhanced, and positive health behaviors can be promoted. Policymakers and healthcare providers should integrate peer support programs into existing maternal health initiatives and fully use social media and community resources to create interactive platforms for maternal and infant knowledge that combine online and offline. By actively promoting peer interaction and experience sharing, these initiatives can maximize the positive role of peer support, increase the utilization rate of health services, and effectively ensure their safety.

## 1 Introduction

Maternal health is a crucial indicator of a country’s economic and social development and is included in the United Nations “Millennium Development Goals” (1). Global differences in social, economic, culture and other factors lead to significant disparities in maternal mortality rates across different countries and regions. Between 2010 and 2022, China’s maternal mortality rate decreased from 30 to 15.7 per 100,000; however, it remained higher in rural areas (16.6 per 100,000) compared to urban areas (14.3 per 100,000) (2). It is estimated that most maternal deaths, along with 25% of infant deaths globally, are closely linked to inadequate maternal health services (3–6). Regional differences seriously impact the effective use of maternal and child health services. In rural areas—especially those that are less developed and more difficult to access—the utilization rate of these services is significantly lower (7). Therefore, improving the effective use of healthcare services in rural areas is a vital issue that must be addressed. This not only jeopardizes rural maternal health but also exacerbates the gap between urban and rural maternal and child health services.

Peer interaction is a behavioral pattern of communication and exchange between individuals of similar ages, social statuses, or ability levels. Its core mechanism lies in the shared meaning systems developed between interactive partners, which are crucial for understanding the learning benefits of peer interaction for individuals (8). In the educational settings, when peers actively engage in discussions and collaborations, they are exposed to diverse viewpoints and thinking modes (9). This exposure effectively stimulates higher-level thinking skills and provides robust support for students’ knowledge acquisition and ability development (10–13). In the fields of medicine and public health, peer interaction significantly influences the health behaviors of patients dealing with various diseases, including chronic conditions and cancer. Patients are generally more receptive to ideas, emotions and information shared by peers due to their similar experiences and demographic characteristics. This interaction enriches their knowledge base regarding health services, empowering them to make more informed health decisions. Additionally, it fosters positive health behaviors, facilitating treatment and recovery (14). For instance, patients dealing with conditions such as acne, stoma, gestational diabetes, and hemiplegia have demonstrated positive outcomes in treatment and rehabilitation through peer interaction (15–18).

In the realm of maternal and child health, PPWs engage with mothers who share similar experiences, community volunteers, and professional maternal and child health guides on essential topics such as pregnancy health care. This interaction can enhance mothers’ knowledge and skills, alleviate psychological stress, and improve the health of their babies (19). In Makwanpur district, Nepal, women’s groups of mothers have raised awareness of the importance of antenatal care through interaction, exchange of experiences and joint involvement in the problem-solving process. This approach has effectively encouraged pregnant women to make more active use of antenatal care services, resulting in a significant reduction in maternal and infant morbidity and mortality (20). A study in rural Malawi showed that sharing health information, such as maternal and child health, through women groups and volunteer peer counseling had a positive effect on improving maternal and child health outcomes (21). Despite the substantial evidence confirming the positive impact of peer interaction in maternal and child health, there is still a lack of in-depth systematic research on its influence specifically regarding the utilization of maternal health services, and the underlying mechanisms remain unclear.

According to social learning theory, individuals can acquire new behavior patterns by observing and imitating others (22). Peer interaction within the maternal groups creates a platform for observation and learning, enabling PPWs to gain health knowledge and behavior patterns through their interactions with experienced mothers. The information-motivation-behavioral skills (IMB) model emphasizes that knowledge is a key factor driving individual behavior change (23). Peer interaction serves as an important channel for disseminating maternal health information, enhancing health awareness, and stimulating intrinsic motivation to utilize health services (24). When motivated, pregnant women are more likely to seek out and engage with these services effectively, leading to increased utilization. A study from Mexico confirmed that rural PPWs sharing information during pregnancy can provide valuable insights into prenatal care (25). This illustrates that knowledge acquisition plays a critical bridging role between peer interaction and service utilization. Therefore, investigating maternal health service knowledge as a mediating variable can aid in analyzing the complex internal mechanisms of how peer interaction affects maternal health service utilization.

In Chinese cities, the advantages of a developed economy, alongside robust medical treatment and educational resources, have fostered various models of peer interaction that support peer support workers (PPWs) in utilizing health services effectively. However, the unique social-economic circumstances in rural areas present significant obstacles to peer interaction, which profoundly impacts the effective use of health services. This study focuses on rural northwest China, aiming to explore the effect of peer interaction on the utilization of maternal health services through the lens of health knowledge growth. By developing an innovative health guidance model, this research seeks to harness the benefits of peer interaction to enhance maternal health awareness, improve compliance with health management practices, and increase the utilization of health services. The findings of this study will provide valuable data to understand the characteristics of rural maternal health care services, inform the formulation of rural family planning health service strategies, promote the balanced development of maternal and child health care in rural areas, and help narrow the gap between urban and rural health services. This research holds significant practical and social value.

## 2 Materials and methods

### 2.1 Data source

This study used baseline data from a survey conducted among PPWs in rural Shaanxi Province. The survey was carried out in two rounds, using identical questionnaires at the same sample sites in March and December 2021. We employed a multi-level cluster random sampling method to identify potential participants. The specific sampling procedure is as follows:

Sample framework establishment: All townships in the sampled counties, excluding the township where the county seat is located, were included in the sample framework. In counties with more than 10 townships, 10 were randomly selected, while in counties with fewer than 10 townships, all available townships were included.

Obtaining a list of pregnant and postpartum women: With the assistance of local health bureaus, we obtained lists of all pregnant and postpartum women in the sampled townships. To manage survey costs, townships with fewer than three pregnant and postpartum women were excluded. Ultimately, the study included 79 townships across 10 counties.

In the March survey, 10 pregnant and postpartum women were randomly selected from each township. If a township had fewer than 10 pregnant and postpartum women, all were included. In the December survey, five women who were within 6 months of pregnancy were randomly selected from each township; in townships with fewer than five pregnant and postpartum women, all were included.

The inclusion criteria for participants were as follows: aged between 18 and 45 years, having lived in the local area for at least 1 year, being within 6 months of pregnancy, and having no history of mental illness or other serious diseases. In total, 821 complete questionnaires were collected, including 592 in March and 229 in December.

### 2.2 Survey method

In this study, we conducted face-to-face interviews with each participant using a questionnaire survey method. These interviews were carried out by rigorously trained enumerators. The questionnaire covered a wide range of topics, including the social-demographic characteristics of the pregnant and postpartum women, basic family information, details about infants, postnatal health check-ups, and family care. It also addressed the utilization and awareness of public services, living conditions, mobility, illness and healthcare services, mental health, e-learning and peer effects, family relationships, and knowledge of health and nutrition during and after pregnancy.

Before each interview, eligible participants were given an informed consent form detailing the study’s objectives, procedures, potential risks, participants’ rights, and privacy protections. To ensure the accuracy and consistency of data collection, we rigorously trained the enumerators and conducted a pilot study with 20 participants before the formal large-scale data collection. During the interviews, all questions were displayed on a tablet computer and asked individually by the enumerators, who recorded the responses. Each interview was conducted in a private setting to avoid interference from other family members and to ensure data independence and accuracy.

### 2.3 Definition and measurement of variables

#### 2.3.1 Dependent variable

The dependent variable in this study was the utilization rate of maternal health services, which includes the maternal system management rate (Y1), prenatal screening rate (Y2), and postpartum visit rate (Y3). In line with the policy requirements outlined in the “Maternal Health Management Service Standard” (26), the maternal system management rate was measured by the question: “Have you established a maternal health manual?” with response options of 0 = “No” and 1 = “Yes.” The prenatal screening rate was assessed with the question: “How many prenatal screenings did you have during this pregnancy?” with options of 0 = less than 5 times and 1 = 5 or more times. The postpartum visit rate was determined by two questions: “Did the hospital staff visit you within 7 days of your newborn’s discharge from the hospital?” The options are 0 = “No,” 1 = “Yes”; “Did the hospital staff visit you within 42 days of your newborn’s discharge?” The options are 0 = “No,” 1 = “Yes.” The weighted results of these two questions were used to calculate the postpartum visit rate, with 0 representing less than 2 visits and 1 representing 2 visits.

#### 2.3.2 Independent variable

The peer category primarily refers to women who are pregnant or have recently given birth (PPWs). Peer interaction is defined as information exchange, emotional support and experience sharing among PPWs. As an independent variable, peer interaction was measured on three dimensions: engagement (social platform), frequency (within 1 month), and quality of interaction (resource utilization richness).

“Do you have any communication groups regarding nutrition and health during pregnancy and infancy (e.g., Social apps like WeChat or QQ)?” Respondent could answer 0 = “No” or 1 = “Yes.” The existence of communication groups serves as an important indicator of modern social connectivity, reflecting the scope of potential peer communication and the number of participants involved. If such a communication group exists, it suggests that a significant number of pregnant women (group members) can freely exchange information, share experiences, and provide emotional support within the group, thereby fostering a peer interaction community.

b. “In the past month, have you discussed your baby’s feeding situation with other parents?” Respondent could answer 0 = “No” or 1 = “Yes.” This question specifically addresses the frequency of peer interaction. By narrowing the time frame to a representative period of the past month, respondents were prompted to reflect on whether they had engaged in discussions about the crucial topic of infant feeding with PPWs. This approach helps to gage the level of active interaction among peers regarding infant care.

c. “How many pregnancy- or parenting-related apps do you have on your mobile phone?” Respondents provided a continuous numerical value as their answer. This question seeks to explore the quality of interaction within peer engagement. The number of apps related to pregnancy or parenting can indicate the various channels through which respondents access information, as well as the richness of resource utilization. These apps typically offer resources such as professional parenting knowledge, practical experience sharing, and opportunities for interaction and communication among participants. Such access can significantly influence the depth and breadth of respondents’ knowledge and the quality of their input during interactions with others, thereby serving as a valuable reference for evaluating the overall quality of peer interaction.

The first two questions involve nominal measurements, primarily aimed at determine whether the respondent has participated in specific types of peer interaction channels and whether such behavior occurred within a designated time frame. For the first question, if the response is “YES,” it is recorded as indicating participation in peer interaction, contributing to the participation scale. Conversely, a “NO” response signifies no recorded participation. For the second question, a higher number of “YES” responses indicated a higher frequency of interaction in the past month. The lower the vice versa. The final question utilizes ratio measurements, which feature explicit zero values and equally spaced units of measure that can be assessed directly. The number of recorded applications serves as a reference value for the quality of interaction. A greater number of apps indicates access to richer, high-quality information resources, likely leading to higher interaction quality. The greater the number, the richer the theoretically available high-quality information resources, and the higher the interaction quality may be. To assess the data suitability for factor analysis, the Kaiser-Meyer-Olkin (KMO) test was conducted, yielding a KMO value of 0.6 (exceeding the acceptable threshold of 0.5), and Bartlett’s test of sphericity was found to be significant. These results affirm that the data are appropriate for factor analysis. Factor scores were calculated to measure the overall level of peer interaction, resulting in a total score for each respondent. The average of all respondents’ factor scores was computed, allowing for the division of participants into two groups: the control group (Control), with scores below the average (X = 0), and the treatment group (PI), with scores above the average (X = 1).

#### 2.3.3 Covariates

The following variables were considered: age, education, pregnancy order, family assets, social medical insurance, medical accessibility, migrant plan (“Do you plan to change your primary residence in the next 6 months?”), and migrant experience (“Have you worked outside before?”).

#### 2.3.4 Mediating variable

Interactions with peers can have complex effects on individual behavior, necessitating consideration of various mediating and moderating variables to fully understand these effects (27). To assess the knowledge of PPWs regarding key health care services from pregnancy through the postpartum period, five questions were posed. These questions covered essential topics such as prenatal visits, postpartum home visits, and physical examinations of infants at different ages. A two-point scoring method was employed, with responses coded as follows: 0 = “I do not know” and 1 = “I know.” The cumulative score across all questions quantifies the level of knowledge, with higher scores indicating a more comprehensive understanding of the relevant health care services. This assessment also facilitates an analysis of the internal relationships between PPWs knowledge, peer interaction, and the utilization of health services.

The questions included:

- Do you know that pregnant and postpartum women need to establish a maternal health manual?
- Do you know that pregnant and postpartum women have five free prenatal screenings?
- Do you know that you need a home visit 7 days after your baby is born?
- Do you know that you need a home visit 42 days after your baby is born?
- Do you know that children need physical examinations at 1 month, 3 months, and half years old?

### 2.4 Statistical analysis

The cross-sectional data from 821 pregnant and postpartum women in rural areas of northwestern China were analyzed using STATA 18.0 software. In the descriptive analysis, categorical variables are presented as frequency (n) and percentage (%), while continuous variables are expressed as mean with standard deviation (SD). Differences between groups were assessed using an independent sample t-test for continuous variables and Chi-square tests for categorical variables. Logistic regression models were employed to obtain coefficients, Z-values, and corresponding confidence intervals (95% CI) to explore the impact of peer interaction on maternal health service utilization.

#### 2.4.1 Logistic regression analysis model

We presented and validated the results of the logistic regression model and compared them with those obtained using the propensity score matching method. The Logistic regression model is specified as follows:


$$\text{Logit}\left(Y\right)=\alpha 1+\beta 1^{*}X+\gamma 1\text{Control}+E$$

In this model, Y represents the dependent variables, including maternal system management rate (Y1), prenatal screening rate (Y2), and postpartum visit rate (Y3); X represents the independent variable, peer interaction (PI). The term “Control” refers to the control variables, which are consistent with the matching variables, including age, education, work company, fixed income, etc.

#### 2.4.2 Propensity score matching method

This study investigated the impact of peer interaction on maternal health service utilization using propensity score matching (PSM). PSM effectively controls for potential confounding factors, creating a data set that approximates “randomized data” (28). “K-nearest Neighbor Matching” was employed with a 1:4 ratio. The matched variables included age, education, pregnancy order, family assets, social medical insurance, medical accessibility, migrant plan, and migrant experience.

#### 2.4.3 Mediating effect model

To examine whether peer interaction (X) positively affects the maternal system management rate (Y1), prenatal screening rate (Y2), and postpartum visit rate (Y3) by enhancing maternal health service knowledge (M), we constructed and tested the following mediation effect model:


$$M=\alpha_{1}+\beta_{1}X+\gamma_{1}\text{Control}+\varepsilon$$

$$Y=\alpha_{2}+\beta_{2}X+\beta_{3}M+\gamma_{2}\text{Control}+\varepsilon$$

## 3 Results

### 3.1 Sample characteristics

This study included 821 PPWs, with their basic demographic characteristics detailed in Table 1. The average age of the respondents was 28.72 years (SD = 3.95), and education level was generally low, with over half (69.43%) having only junior high school and below. Most PPWs (90.74%) did not have a work company, and 92.20% lacked a fixed income. The average family size was approximately seven people, and the average total family income last year was 67,900 RMB. Among the participants, 13.89% were from registered family assets, and 34.47% were first-time mothers. The average distance from their residence to the nearest birthing hospital was 19.83 km. Social medical insurance (new rural cooperative medical insurance) was held by 88.92% of the respondents. Most PPWs (73.33%) had experience working outside the home, and 8.65% planned to change their residence within the next 6 months. The systematic management rate among the sample was 97.93%, the prenatal screening rate (5 times or more) was 58.10%, and the postpartum visit rate (2 times) was 53.47%.

**Table 1:** Demographic characteristics.

Variables	Description	Mean ± standard deviation/percentage
Panel A: individual characteristics		
Age	Mean age	28.72 ± 3.95
Education		
Junior school and below	Percentage of junior school and below	69.47%
High school and above	Percentage of high school and above	30.53%
Work company	Percentage of company with work	9.26%
Fixed income	Percentage of a fixed income	7.80%
Panel B: family characteristics		
Family population	Mean family population	6.90 ± 0.82
Pregnancy order	Percentage of first pregnancy or first-born child	34.47%
Household income	Average annual household income (RMB 10,000)	6.79 ± 4.13
<3		10.96%
3 ≤ Household Income <6		46.53%
≥6		42.51%
Family assets	Percentage of family assets below the local average	13.89%
Panel C: social characteristics		
Social medical insurance	Percentage of new rural cooperative medical insurance	88.92%
Medical accessibility (km)	Average distance from a formal medical facility	19.83 ± 15.05
Migrant plan	Percentage of migrant plans within the next 6 months	8.65%
Migrant experience	Percentage of worked outside the home in the past	73.33%
Panel D: Maternal health service utilization		
Maternal system management rate	Percentage of maternal system management	97.93%
Prenatal screening rate	Percentage of prenatal screening (≥5 times)	58.10%
Postpartum visit rate	Percentage of postpartum visit (2 times)	53.47%

### 3.2 Propensity score matching

Propensity score matching (PSM) is an effective method to reduce the influence of confounding factors in observational studies. Through propensity score matching, logistic regression estimation is used to make the treatment group (PI) and control group (Control) as similar as possible on multiple covariates, making subsequent estimates of corresponding treatments more accurate. This study screened for factors affecting maternal health service utilization (Y1, Y2, and Y3). Age, education, pregnancy order, family assets, social medical insurance, medical accessibility, migrant plan, and migrant experience factors were used as confounding factors to match the samples.

Before matching, the mean age in PI was 28.308, and that in Control was 29.366, with a deviation of −26.900, with a deviation reduction of 98.7%, and the T-test result was significant (*p* < 0.01). This indicates that there is a large difference in age between PI and Control when there is no matching, and this difference is greatly reduced after matching, indicating that matching balances the confounding factor of age to some extent. For pregnancy order, the bias before matching was 16.200, with an 89.3% reduction in bias and T-test significance (*p* < 0.05). This suggests that matching this variable is a good balance, and pregnancy order may have some influence on the results. In addition, the deviation of the variable medical accessibility before matching is −20.800; the deviation is reduced by 99.2%, and the *p*-value of the T-test is less than 0.01, which is of significant significance. This indicates that the difference between PI and Control before matching is large, and the difference is greatly reduced after matching, and this variable may have an important impact on the results.

In summary, PSM balances the differences between PI and Control among individual covariates to varying degrees. Among them, age, pregnancy order, and medical accessibility had significant differences before matching and may have important effects on the results, but the differences decreased after matching. However, variables such as education, family assets, social medical insurance, migrant plan, and migrant experience have relatively little impact on the results before and after matching (Table 2).

**Table 2:** Standardized deviations for each variable before and after matching.

Variable	Unmatched	Mean		% Reduction		*T*-test	
	Matched	PI	Control	% Bias	|Bias|	*t*	*p* > |t|
Age	U	28.308	29.366	−26.900	98.7	−3.770	0.000***
	M	28.366	28.352	0.400		0.060	0.955
Education	U	3.915	3.854	4.300	6.0	0.600	0.549
	M	3.924	3.982	−4.000		−0.630	0.530
Pregnancy order	U	0.374	0.298	16.200	89.3	2.250	0.025**
	M	0.370	0.362	1.700		0.270	0.791
Family assets	U	0.130	0.155	−7.400	80.1	−1.040	0.301
	M	0.131	0.126	1.500		0.240	0.811
Social Medical insurance	U	0.895	0.888	2.100	228.4	−0.070	0.944
	M	0.894	0.872	6.900		0.070	0.943
Medical accessibility	U	18.564	21.725	−20.800	99.2	−2.940	0.003***
	M	18.696	18.721	−0.200		−0.030	0.978
Migrant plan	U	0.089	0.084	1.900	331.1	0.260	0.796
	M	0.088	0.110	−8.000		−1.180	0.240
Migrant experience	U	0.749	0.714	7.800	86.7	1.100	0.273
	M	0.746	0.742	1.000		0.160	0.869

*p values in bold are statistically significant, ***p < 0.01; **p < 0.05; *p < 0.1.

Table 3 shows the influence of variable X before PSM on Y1, Y2, and Y3. The regression coefficient of variable X to Y1 is 0.851*(0.488), and the 95% confidence interval is [−0.105,1.808]. This indicates that when other factors are controlled, there is a positive correlation between variable X and Y1, and it is significant at the level of *p* < 0.1. The regression coefficient of variable X to Y2 is 0.475**(0.201), and the 95% confidence interval is [0.082,0.868]. It is significant at the *p* < 0.05 level, indicating that there is a relatively obvious positive correlation between variables X and Y2. The regression coefficient of variable X to Y3 is 0.448***(0.110), and the 95% confidence interval is [0.231,0.664]. It is highly significant at the *p* < 0.01 level, indicating a strong positive correlation between the variables X and Y3.

**Table 3:** Effect of peer interaction before PSM on maternal health service utilization.

Variables	Y1		Y2		Y3	
	*β* Coefficient (Std. err.)	95% Confidence interval	*β* Coefficient (Std. err.)	95% Confidence interval	*β* Coefficient (Std. err.)	95%Confidence interval
X	0.851*(0.488)	−0.105,1.808	0.475**(0.201)	0.082,0.868	0.448***(0.110)	0.231,0.664
Age	−0.041 (0.072)	−0.182,0.101	0.017 (0.021)	−0.025,0.058	0.003 (0.016)	−0.028,0.034
Education	1.297***(0.497)	0.322,2.271	−0.035 (0.078)	−0.178,0.127	0.043 (0.034)	0.024,0.110
Pregnancy order	−0.134 (0.387)	−0.892,0.625	−0.202*(0.120)	−0.438,0.034	−0.376***(0.104)	−0.579,-0.173
Family assets	−0.103 (0.427)	−0.939,0.733	−0.329 (0.384)	−1.083,0.424	−0.109 (0.102)	−0.308,0.090
Social medical insurance	1.932***(0.533)	0.848,3.016	−0.485 (0.517)	−1.499,0.529	−0.203 (0.343)	−0.875,0.469
Medical Accessibility	−0.001 (0.016)	−0.032,0.031	0.022**(0.010)	0.003,0.041	−0.010 (0.009)	−0.028,0.008
Migrant plan	0.312 (0.692)	−1.044,1.668	−0.013 (0.243)	−0.490,0.464	−0.652***(0.210)	−1.063, −0.242
Migrant experience	−0.631 (0.705)	−2.014,0.751	−0.063 (0.142)	−0.340,0.215	−0.171 (0.252)	−0.666,0.324
Constant	−1.471 (1.726)	−4.855,1.912	0.127 (0.852)	−1.543,1.797	−0.404 (0.505)	−1.393,0.586
*R* ^2^	0.147		0.027		0.019	
Observations	662		662		662	

*p values in bold are statistically significant, ***p < 0.01, ** *p* < 0.05, * *p* < 0.1.

Table 4 shows the influence of variable X on Y1, Y2, and Y3 after PSM. The regression coefficient of variable X on Y1 is 0.856*(0.486), and the 95% confidence interval is [−0.097,1.809], which indicates that there is a positive correlation between variable X and Y1 when other factors are controlled, and it is significant at the level of *p* < 0.1. The regression coefficient of variable X to Y2 is 0.439**(0.211), and the 95% confidence interval is [0.026,0.853]. It is significant at the *p* < 0.05 level, indicating that there is a relatively obvious positive correlation between variables X and Y2. The regression coefficient of variable X on Y3 is 0.479***(0.121), and the 95% confidence interval is [0.241,0.716], which is highly significant at the level of *p* < 0.01, indicating that there is a strong positive correlation between variables X and Y3.

**Table 4:** Effect of peer interaction after PSM on maternal health service utilization.

Variables	Y1		Y2		Y3	
	*β* Coefficient (Std. err.)	95% confidence interval	*β* Coefficient (Std. err.)	95% confidence interval	*β* Coefficient (Std. err.)	95% confidence interval
X	0.856* (0.486)	−0.097,1.809	0.439** (0.211)	0.026,0.853	0.479*** (0.121)	0.241,0.716
Age	−0.041 (0.072)	−0.182,0.101	0.021 (0.018)	−0.014,0.057	−0.001 (0.015)	−0.032,0.029
Education	1.300*** (0.493)	0.334,2.266	−0.021 (0.083)	−0.183,0.141	0.038 (0.033)	−0.026,0.102
Pregnancy order	−0.145 (0.398)	−0.925,0.636	−0.215* (0.126)	−0.461,0.032	−0.360*** (0.101)	−0.559,-0.162
Family assets	−0.125 (0.433)	−0.974,0.724	−0.280 (0.391)	−1.045,0.485	−0.151 (0.125)	−0.395,0.093
Social medical insurance	1.911***(0.586)	0.799,3.024	−0.472 (0.523)	−1.496,0.553	−0.213 (0.359)	−0.916,0.489
Medical accessibility	−0.001 (0.016)	−0.033,0.030	0.024** (0.010)	0.004,0.043	−0.011 (0.010)	−0.031,0.009
Migrant plan	0.327 (0.681)	−1.008,1.661	−0.011 (0.251)	−0.502,0.481	−0.655*** (0.208)	−1.063,-0.247
Migrant experience	−0.600 (0.709)	−1.989,0.789	−0.096 (0.171)	−0.432,0.240	−0.148 (0.278)	−0.693,0.397
Constant	−1.494 (1.731)	−4.887,1.900	0.008 (0.775)	−1.511,1.528	−0.297 (0.514)	−1.305,0.712
*R* ^2^	0.148		0.029		0.020	
Observations	658		658		658	

*p values in bold are statistically significant; ***p < 0.01; **p < 0.05; *p < 0.1.

In summary, PSM plays a role in controlling confounding factors. It proves that there is a significant positive correlation between X and dependent variables (Y1, Y2, and Y3), and these relationships remain relatively stable in statistical significance and strength before and after matching.

### 3.3 The mediating effect of maternal health service knowledge

In Table 3, the results of the mediating effect of stepwise regression show that in the regression model that only considers independent variables X and dependent variables (Y1, Y2, and Y3), the coefficient of X to Y1 is 0.851*(0.488), and the 95% confidence interval is [−0.105,1.808]. The coefficient of Y2 is 0.475**(0.201), and the 95% confidence interval is [0.082,0.868]. The coefficient of Y3 is 0.448***(0.110), and the 95% confidence interval is [0.231,0.664]. This suggests that peer interaction (X) has a direct positive effect on the maternal system management rate (Y1), prenatal screening rate (Y2), and postpartum visit rate (Y3).

When the regression model of intermediary variable M and independent variable X is established, the coefficient of X and M is 0.023***(0.004), indicating that X has a certain influence on maternal health service knowledge (M) (Table 5). In the regression model considering independent variable X, mediating variable M and dependent variables (Y1, Y2, and Y3) at the same time, the coefficient of X to Y1 is 0.783*(0.467), and the 95% confidence interval is [−0.133,1.699]. The coefficient of Y2 is 0.480**(0.206), and the 95% confidence interval is [0.077,0.883]. The coefficient of Y3 is 0.422***(0.132), and the 95% confidence interval is [0.160,0.683]. Meanwhile, the coefficient from M to Y1 is 2.380***(0.857), and the 95% confidence interval is [0.699,4.060]. The coefficient for Y2 is −0.203(0.319), and the 95% confidence interval is [−0.828,0.422]. The coefficient for Y3 is 3.751***(1.283), and the 95% confidence interval is [1.237, 6.265].

**Table 5:** The mediating effect of maternal health service knowledge.

	Model 2		Model 3		Model 3		Model 3	
Var	M		Y1		Y2		Y3	
	*β* Coefficient (Std. err.)	95% confidence interval	*β* Coefficient (Std. err.)	95% confidence interval	*β* Coefficient (Std. err.)	95% confidence interval	*β* Coefficient (Std. err.)	95% confidence interval
X	0.023*** (0.004)	0.012,0.033	0.783* (0.467)	−0.133,1.699	0.480** (0.206)	0.077,0.883	0.422*** (0.132)	0.160,0.683
M			2.380*** (0.857)	0.699,4.060	−0.203 (0.319)	−0.828,0.422	3.751*** (1.283)	1.237,6.265
Control variables	YES							
Constant	0.407** (0.133)	0.037,0.776	−2.100 (1.868)	−5.761,1.561	−0.208 (0.951)	−1.657,2.073	−1.954 (0.526)	−2.985,-0.924
*R* ^2^	0.017		0.163		0.027		0.082	
Observations	662		662		662		662	

*p values in bold are statistically significant, ***p < 0.01; **p < 0.05; *p < 0.1.

The characteristics of different dependent variables and the role of peer interaction and maternal service knowledge in different links are different. Variable X has different degrees of positive correlation with different dependent variables Y1, Y2, and Y3, and the level of significance is different. The intermediate variable M has a strong positive correlation with Y1 and Y3, while the relationship with Y2 is not obvious. This may indicate that the influence of variable X on Y1 and Y3 is partly achieved through the intermediary variable M, while there may be other influence mechanisms or variables at work for Y2.

Maternal service knowledge has a strong positive correlation with maternal system management rate (Y1) and postpartum visit rate (Y3), which indicates that peer interaction plays a key role in knowledge dissemination and sharing. Peer interaction of relevant knowledge can enhance the attention and awareness of maternal system management, promote participation, and realize partial influence transmission. In terms of postpartum visits, pregnant women who understand the importance of postpartum visits are more likely to cooperate with the visits, and peer sharing of experience and knowledge can enhance the identification and willingness to participate in the visits. As for prenatal screening rate (Y2), because it mainly involves the selection of specific medical examination items, pregnant women are more influenced by doctors’ recommendations, their health status cognition, and family factors when deciding whether to undergo prenatal screening and peer interaction has relatively little influence. This is because prenatal screening often requires specialized medical knowledge to explain its importance, and peers may not have accurate enough expertise to strongly influence others’ decisions, making it difficult for maternal service knowledge to play a significant mediating role in this process.

## 4 Discussion

This study analyzed the cross-sectional data of 821 PPWs in rural northwest China, employing the propensity score matching (PSM) method to explore the impact of peer interaction on maternal health service utilization. It also empirically evaluated the mediating role of maternal health service knowledge. The results indicate that peer interaction significantly enhances the utilization rate of maternal health services in rural areas, as evidenced by improvements in maternal health service system management, prenatal screenings, and postnatal visits. Additionally, the research confirms that peer interaction plays a critical role in improving maternal health behaviors by increasing knowledge of maternal health services. These findings provide valuable empirical insights into the underlying mechanisms at play and hold significant implications for the design of maternal health service interventions in rural areas.

From the sample characteristics, the prenatal health screening rate (≥5 times) among PPWs in this study was 58.10%, significantly lower than the national average of 88.7% in 2022. The postpartum visit rate (2 times) also stood at was 53.47%, well below the 2022 average of 96.5% (2). Most have a low level of education, only junior high school and below, resulting in a lack of awareness of health services and a lack of awareness of initiative. Most people have unstable employment, fixed incomes and heavy economic burdens, which limit their ability to access medical resources, such as many women forgoing or delaying prenatal screening and postnatal visits for financial reasons. Nearly 9 per cent plan to relocate, and high mobility creates an unstable environment and reduces the frequency of use of health services, well below the national level. The interaction of these factors leads to the low level of utilization of maternal health services in rural northwest China, while the unstable environment impedes access to services for pregnant women, affects peer communication and experience sharing, and weakens the effect of peer interaction, which in turn affects the decision-making and behavior of health service utilization.

Knowledge and service utilization through peer interaction, targeted strategies are crucial. Given the low levels of education in certain communities, it is essential to consider both the methods and content used for knowledge transfer during peer interactions (29). Traditional methods have shown limited results; however, they can still facilitate peer support. Individuals who possess cultural or experiential knowledge can simplify complex medical information into relatable examples, thereby increasing awareness among those with lower educational backgrounds. For pregnant individuals facing financial challenges, sharing affordable healthcare strategies and utilizing local free or low-cost medical resources can be beneficial. Recognizing the barriers posed by high mobility among peer groups, establishing an online platform through modern media can enhance communication and broaden interaction visibility. Organizing knowledge-sharing sessions via online video conferencing, wherein experts or experienced individuals can address questions, represents a promising approach (30). For example, organize knowledge sharing sessions through online video conferencing, and invite experts or experienced people to answer questions. In addition, hospitals and communities should actively explore innovative health education techniques. This can include investments in online resources, the production of various educational videos, and the creation of online consultation and peer interaction groups. In these groups, both medical and community staff can facilitate discussions and provide answers to enhance the quality of peer interactions (31).

Peer interaction plays a vital role in prenatal screening, albeit its direct impact may appear small. It significantly contributes to strengthening maternal adherence to medical recommendations. When individuals share their experiences following these recommendations and screenings, it fosters a supportive environment that encourages others to do the same. During postpartum visits, partners can motivate and remind each other about the importance of follow-up care. By sharing experiences and precautions, they can raise collective awareness about the necessity of return visits, potentially improving attendance rates. To effectively enhance maternal health care services in rural areas, relevant government departments should consider the following measures: providing financial support can alleviate the economic burden on pregnant individuals, enabling them to access essential prenatal and postpartum care without the fear of incurring insurmountable costs. Improving rural medical facilities and increasing the accessibility of health services are crucial steps to ensure that all expectant mothers receive the necessary care. Policy initiatives should encourage the involvement of social organizations and volunteers. These groups can enrich peer interaction resources and forms, thereby offering emotional support, information sharing, and companionship services for pregnant individuals in rural areas.

In conclusion, the results of this study provide an important basis for improving the utilization of maternal health services in rural areas of northwest China. By taking full advantage of the power of peer interaction and taking targeted measures to improve maternal health knowledge, it is expected to improve the overall level, reduce adverse pregnancy outcomes, and safeguard maternal and child health. Future research can further explore how to accurately promote peer interaction and resource integration to maximize service utilization.

## 5 Conclusion

Maternal health management is crucial for effectively reducing maternal and perinatal mortality rates. Despite substantial efforts by the State and Government to ensure maternal health, the utilization of maternal health services in rural areas remains insufficient. Traditional education models often fail to engage rural pregnant and postpartum women with lower education levels meaningfully. The implementation of the Healthy China strategy offers a new perspective on innovating maternal health management models. By adopting the “Knowledge, Attitude, Practice” (KAP) framework from health education theory, peer interaction can significantly enhance pregnant and postpartum women’s understanding of health services, promote positive health behaviors, and foster a greater sense of participation.

Policymakers and healthcare providers should integrate peer interaction programs into current maternal health interventions. Leveraging social media and community resources can create both online and offline platforms for sharing pregnancy-related knowledge. Primary health services can group women by similar cultural backgrounds and age to facilitate experience sharing and support. These strategies not only enhance the positive impact of peer support, encourage self-management, and improve compliance but also boost health service utilization, safeguard maternal and child health, and elevate overall population quality.

## Data Availability

The datasets used and/or analyzed during the current study are available from the corresponding author on reasonable request.
